# Social return on investment economic evaluation of supportive care for lung cancer patients in acute care settings in Australia

**DOI:** 10.1186/s12913-022-08800-x

**Published:** 2022-11-23

**Authors:** Amelia Hyatt, Holly Chung, Ruth Aston, Karla Gough, Meinir Krishnasamy

**Affiliations:** 1 Department of Health Services Research, Peter MacCallum Cancer Centre, 305 Grattan Street Parkville, Melbourne, Australia; 2 Sir Peter MacCallum Department of Oncology, The University of Melbourne, VIC 3010 Melbourne, Australia; 3 Academic Nursing Unit, Peter MacCallum Cancer Centre, Melbourne, Australia; 4 Centre for Program Evaluation, Melbourne Graduate School of Education, The University of Melbourne, VIC 3010 Melbourne, Australia; 5 Department of Nursing, Faculty of Medicine, Dentistry, and Health Sciences, The University of Melbourne, 3052 Melbourne, Australia; 6 Victorian Comprehensive Cancer Centre Alliance, VIC 3010 Melbourne, Australia

**Keywords:** Cancer, Social return on investment, Supportive care, Lung cancer, Economic evaluation

## Abstract

**Background:**

Unmanaged consequences of cancer and its treatment are high among patients with lung cancer and their informal carers, resulting in avoidable healthcare use and financial burden. Provision of cancer supportive care addressing the impacts of cancer and its treatment has demonstrated efficacy in mitigating these consequences; however, globally, there is a lack of investment in these services. Paucity of robust economic evidence regarding benefit of cancer supportive care has limited policy impact and allocation of resources. This study therefore utilised a Social Return on Investment (SROI) methodology to conduct a forecast evaluation of lung cancer supportive care services, to ascertain potential social value and return on investment.

**Methods:**

An SROI economic evaluation model was developed using qualitative stakeholder consultations synthesised with published evidence to develop the inputs, outcomes and financial value associated with the delivery of a hypothetical model of quality lung cancer supportive care services over a one and five year period. SROI ratios were generated to determine the social value and cost savings associated per every $1AUD invested in cancer supportive care for both the healthcare system and patients. Deadweight, drop off and attribution were calculated, and sensitivity analysis was performed to confirm the stability of the model.

**Results:**

The value generated from modelled supportive care service investments in a one-year period resulted in an SROI ratio of 1:9; that is, for every dollar invested in supportive care, AUD$9.00 social return is obtained when savings to the healthcare system and benefits to the patients are combined. At five-years, these same investments resulted in greater cumulative value generated for both the patient and the healthcare system, with a SROI ratio of 1:11.

**Conclusion:**

Our study provides strong evidence for policy makers, clinicians and consumers to advocate for further investment in cancer supportive care, as demonstrated cost savings could be achieved through implementation of the proposed supportive care service model, with these accruing over a five-year period. The SROI model provides a comprehensive framework detailing supportive care services and the health workforce necessary to achieve value-based outcomes for patients and the healthcare system.

**Supplementary Information:**

The online version contains supplementary material available at 10.1186/s12913-022-08800-x.

## Background

Cancer incidence and mortality is increasing globally with an estimated 28.4 million new cancer cases projected to occur in 2040, a 47% increase from 2020 [1]. Lung cancer is the second most commonly diagnosed cancer, and the current leading cause of cancer deaths worldwide [1]. Innovations in treatment, particularly in high income countries, are improving survival opportunities, with individuals diagnosed with lung cancer now living longer [2]. However, individual, social and healthcare system costs incurred as a consequence of lung cancer morbidity and mortality are significant, and increasing [3, 4]. Importantly, economic impacts of lung cancer are largely the result of unmet patient and family caregiver need, in managing the effects of the disease and its treatments [4].

Cancer supportive care refers to the integrated, multidisciplinary suite of services provided to people diagnosed with cancer to address complex needs associated with cancer and its treatment [5]. Provision of cancer supportive care addresses the informational, physical, financial, social, and psychological consequences of cancer, which when unaddressed, impact health outcomes and quality of life [6]. Despite a plethora of evidence demonstrating significant clinician, patient-reported and healthcare system benefits associated with timely access to supportive care services; subsequent integration into tertiary healthcare services has been limited [7, 8]. Mapping of current supportive care service provision in Australia revealed that service implementation is non-standard, highly variable, under-evaluated, unaligned to patient need, and of inequitable access [8]. Patients affected by lung cancer continue to experience and report high unmet need, indicating the urgency of addressing this gap in care [9].

Increasing scarcity of public resources has led governments and policy-makers to seek robust economic assessments of healthcare interventions to assess value-for-money [10]. However, paucity of economic data associated with quality cancer supportive care has limited policy initiatives and investment to date. Traditional economic analysis approaches such as cost-effectiveness and cost-utility are useful. However, with limited economic data or economic evaluations of alternatives to supportive care, application of these analyses are limited in their ability to forecast the benefits that can result from health interventions, such as those provided by cancer supportive care [11]. Social Return on Investment (SROI), is an expanded form of cost-benefit analysis that seeks to capture the social, economic, and environmental impacts of interventions, as well as acknowledge potential negative effects of services [10]. The broader concept of value captured by SROI methodology provides a robust framework to conduct a forecast evaluation to determine the value and return on investment for an evidence-based model of quality cancer supportive care services for tertiary healthcare settings [10, 12]. Importantly SROI allows the comprehensive impact of health interventions to be captured to inform evidence-based policy and investment strategies.

This study utilised the SROI methodology to achieve two objectives: first, to describe a stakeholder, and evidence-informed potential model of quality cancer supportive care for people affected by lung cancer in tertiary healthcare settings; and second, to conduct a forecast evaluation of the model to ascertain its potential social value and return on investment.

## Methods

SROI methodology comprises both a framework to gather data to form a prospective program model, and conduct a health economic analysis. A mixed methods approach inclusive of semi-structured interviews, narrative literature review and theory of change was employed to generate and gather all relevant data to build the model. Then, quantitative SROI economic analysis calculations were employed to ascertain return on investment from the application of the model. The 0-12-point scoring index utilised to assess SROI quality in previous systematic reviews and meta-analyses was employed as a checklist to guide reporting against all 12 indices of quality [11, 13].

Lung cancer services at two tertiary, public hospitals in metropolitan Melbourne (one with supportive care screening policy and one without) were selected as representative ‘case examples’ to ascertain value associated with the integration of quality cancer supportive care into routine service provision. This project was reviewed and approved by Peter MacCallum Cancer Centre Human Research Ethics Committee (HREC) (multi-site approval number: HREC/66771/PMCC).

### Data collection

Social return on investment methodology employs six stages (Table 1), underpinned by eight key principles: involve stakeholders; understand what changes; value the things that matter; only include what is material; do not over claim; be transparent; verify the result, and be responsive [12]. The first four stages outline the steps required to develop the SROI model. These stages involve iterative data collection and synthesis, as inputs, outputs, and outcomes are established, and associated costing data are confirmed. The finalised model is then utilised to conduct the SROI economic analysis in Stage 5.

**Table 1:** SROI stages, activities, and outputs

SROI Stages	Activities	Outputs
**Stage 1: ** **Establishing Scope and Identifying Stakeholders**	• Scoping review 1 and stakeholder consultation to determine key supportive care needs • Stakeholder identification and engagement methods determined	• Supportive care scope and priorities for people with lung cancer defined • SROI model scope determined • HREC application and study protocol approved
**Stage 2: ** **Mapping Inputs and Outcomes**	• Scoping review 2 evidence for relevant inputs and outcomes • Stakeholder consultation data analysed to identify stakeholder-informed themes • Evidence Synthesis to triangulate evidence with local context	• Inputs and outcomes identified and grounded in evidence • Consensus Map created to document evidence synthesis • Theory of Change illustrates mechanisms of action
**Stage 3: ** **Evidencing and Valuing Inputs and Outcomes**	• Cost-ingredient approach used to value inputs • Continued evidence synthesis of stakeholder consultation data and published evidence used to assign indicator and/or proxies to outcomes	• Inputs are valued • Indicators assigned to outcomes; financial proxies assigned to indicators • Value Map created
**Stage 4: ** **Establishing Impact**	• Outcome indicators and supportive care inputs assigned dollar value per capita • Key scenarios and assumptions identified	• Per capita value adjusted for: deadweight, attribution and drop off • Sensitivity analysis performed
**Stage 5: ** **Calculating the SROI**	• Combined value generated calculated for 1- and 5-year cohorts • Healthcare system value calculated for 1- and 5-year cohorts Patient value calculated for 1- and 5-year cohorts	• SROI ratios/value generated interpreted • Limitations outlined

Literature considered for this study comprised published journal articles reporting the symptomatology, needs, experiences, and perspectives of lung cancer patients. Journal articles included in this review included quantitative and qualitative studies, review articles, and synthesis studies (including meta-analyses, systematic reviews, and meta-synthesis) papers, which were identified through searches on Google Scholar between 2nd October 2020 and 13 April 2021. Search terms included the following terms and variations thereof: “lung cancer”, “patient experiences”, “supportive care” “needs”, “burden” with the use of AND/OR Boolean operators. Back- and forwards citation chasing was utilised for appropriate papers. Only papers written in English were included. Emphasis was placed on qualitative studies reporting on the experiences of lung cancer patients. Qualitative studies investigating the experiences of carers, patient/carer dyads, and health professionals treating lung cancer patients were excluded. While initial searches included literature from 2000 onwards to identify seminal papers in the field, subsequent searches were restricted to literature published from 2016 onwards, acknowledging substantial advances in lung cancer treatment which occurred with arrival of immunotherapy [14].

#### Stage 1: establishing scope and identifying stakeholders

Stage 1 comprised preliminary decisions regarding project scope necessary for development of the SROI model. Scope parameters included: time forecasts, stakeholders, and essential areas of patient supportive care need. These parameters were essential to clearly document five key assumptions underpinning the tertiary healthcare service model of integrated supportive care service delivery for lung cancer patients (Table 2). The decision-making process underpinning scope parameters was initially informed by a comprehensive literature review; then, triangulated with stakeholder consultation data in Stage 2 to ensure transparency and rigour.

**Table 2:** Key assumptions underpinning the SROI model

Assumption 1:	Costs of supportive care inputs were calculated for face-to-face care delivery only. It was, however, acknowledged that some supportive care will be delivered via telehealth or via web-based resources in the applied scenarios. Face-to-face delivery of care requires the highest investment, and therefore using this approach means that SROI ratios and associated value generated are conservative.
Assumption 2:	Supportive care will be delivered over a five-year period throughout treatment cycles for lung cancer patients. The SROI analysis assumed that supportive care continues to be delivered to patients over a one- and five-year period alike. A minimum of supportive care inputs, delivered over one year was assumed, acknowledging that it is likely that supportive care inputs will be clustered around treatment cycles.
Assumption 3:	Maximum value is assumed for the healthcare system, patients and their families as a result of access to quality cancer supportive care. The SROI analysis was undertaken based on the assumption that all patients receive high quality supportive care. In other words, variation in care quality was not considered in the SROI. Based on this assumption, social value estimates represent maximum value generated from receipt of quality cancer supportive care. Further, it was assumed that patients have high supportive cancer care needs, therefore require comprehensive cancer supportive care.
Assumption 4:	Patients that experience a higher quality of life as a consequence of receiving quality supportive care, will engage in activities associated with better quality of life, such as physical activity. The SROI analysis assumed that patients that experience a higher quality of life will engage in activities like accessing gyms, sports and recreational facilities, and other social activities with friends and family. In this scenario, the total value of patient benefit is reduced due to the cost of engaging in these activities, and therefore is a more conservative estimate of the value generated to patients.
Assumption 5:	Patients live beyond the one- and five-year periods. The SROI analysis assumed that within each time-forecast, some patients survived beyond one and five years.

Time forecasts were informed by standardised reporting metrics for lung cancer and lung cancer survival rates. On average in Australia, only 45% of people diagnosed with lung cancer survive one year post-diagnosis, and 20% survive five years. [15]. Therefore, two time-forecasts were defined: people diagnosed with lung cancer with a prognosis of < 1 year, and people diagnosed with lung cancer with.

a prognosis of > 5 years. By including the two time-forecasts the poor survival rates associated with lung cancer were acknowledged in forecasting the social value of supportive care, while also capturing the full suite of supportive care services that complement palliative, radical palliative, and curative treatment pathways.

Prevalent and burdensome needs reported by patients with lung cancer were identified through the first of two comprehensive scoping reviews undertaken to generate evidence summaries which underpin the SROI model (supplemental materials). Five cancer supportive care needs and related activities were included in the SROI model: screening; equitable and coordinated care; information; financial toxicity; and anxiety and depression. These needs informed the subsequent determination of activity, input, outcome, and valuation components of the SROI model (Table 1).

Three key primary stakeholder groups were then identified: people diagnosed with lung cancer; healthcare professionals involved in the care of people with lung cancer; and healthcare service managers involved in the implementation of policy, strategy, and operational delivery of oncology and/or supportive care services.

#### Stage 2: mapping inputs and outcomes

Stakeholder consultations were undertaken to gather data for SROI Stages 2–4 (Table 1), via telephone or video-conferencing, utilising a semi-structured qualitative interview schedule to guide data collection. De-identified interview transcripts were uploaded into a qualitative data management software program (NVivo 12), and a coding frame developed to deductively identify stakeholder descriptions of inputs, outcomes, and value associated with the delivery of valued services. A total of 23 people with lung cancer and 11 health professionals and healthcare service managers across two health services (Table 3) took part in the consultations which ran on average for 49 min (range: 22 to 65 min). All stakeholders provided informed consent.

**Table 3:** Stakeholder Demographics

**Patient Stakeholders**			**Total** (***n* = 23)**			**Health Service A** (***n* = 15)**		**Health Service B** **(*n* = 8)**		
**Age (years)**										
Mean, SD			67	8		67	8	67	10	
Min, Max			53	82		57	82	53	77	
**Time Since Diagnosis (months)**^**a**^										
Mean, SD			12	7		11	7	15	7	
Min, Max			4	31		5	31	4	24	
**Gender**			***n***	**%**		***n***	**%**	***n***	**%**	
Male			10	43		6	40	4	50	
Female			13	57		9	60	4	50	
**English first language**										
Yes			13	57		9	60	4	50	
No			10	43		6	40	4	50	
**Marital Status**^**b**^										
Single			4	17		2	13	2	25	
Married/De Facto			10	43		6	40	4	50	
Separated/Divorced			7	30		6	40	1	13	
Widowed			2	9		1	7	1	13	
**Aboriginal and/or Torres Strait Islander**										
Yes			0	0		0	0	0	0	
No			23	100		15	100	8	100	
**Current Employment Status**										
Employed (full time/part time)			1	4		1	7	0	0	
Not in Paid Employment			10	43		6	40	4	50	
Taking Sick or Personal Leave			0	0		0	0	0	0	
Retired			12	53		8	53	4	50	
**Highest Level of Education**^**b**^										
Partial Secondary			16	70		10	67	7	88	
Completed Secondary (year 12)			3	12		2	13	0	0	
Trade/TAFE			2	9		1	7	1	13	
University			2	9		2	13	0	0	
**Diagnosis**										
Lung Cancer (did not specify)			12	52		9	60	5	62	
NSCLC Stage III/IV			11	47		5	34	3	38	
**Healthcare Professionals and Service Managers**			**Total** ***n* = 11**			**Health Service A** ***n* = 5**		**Western Health** ***n* = 6**		
**Time at Healthcare service (years)**										
Mean, SD			15		10	16	11	12	5	
Min, Max			6		37	6	37	8	20	
**Gender**			***n***		**%**	***n***	**%**	n	%	
Male			4		36	2	40	2	33	
Female			7		64	3	60	4	67	
**Role Category**										
Doctor			5		46	1	20	4	66	
Nurse			4		36	3	60	1	17	
Allied Health			2		18	1	20	1	17	
**Stakeholder representation**										
Healthcare Professional			8		73	4	80	4	67	
Healthcare Service Manager			3		27	1	20	2	33	

^a^n=22

^b^due to rounding, percentages may not add up to 100

Concurrently, a second scoping review was conducted to establish: the most common activities associated with services provided (inputs) by hospitals to address identified supportive care needs experienced by lung cancer patients (screening; equitable and coordinated care; information; financial toxicity; and anxiety and depression); and evidence-based outcomes associated with delivery of these inputs. Published evidence was compared with evidence from stakeholder consultations, and where there was alignment, a supportive care input and/or outcome statement was articulated, informed by theory of change analysis [16]. For each statement, an identification of whether evidence available implied a direct association or causal relationship between ‘necessary’ supportive care activities (inputs) and confirmed benefits (outcomes) was undertaken. These ‘evidence syntheses’ were applied across all five supportive care need areas included in the SROI model for all three stakeholder groups, resulting in a total of 43 inputs, and 34 outcomes generated across both one-year and five-year time forecasts. Data from Stages 1–4 were documented in a spreadsheet known as the ‘value map’ in SROI terminology (supplemental materials).

#### Stage 3: evidencing and valuing inputs and outcomes

Inputs: In this stage, the direct cost investment required for each necessary input was investigated (valuation). As this SROI study applied a theoretical model to forecast return on investment of future delivery of supportive care, primary economic data to calculate input costs were not available. To address this, a cost-ingredient approach was undertaken where each “input” was assigned a market value, with assumptions based on stakeholder consultations in conjunction with existing published data for the reference year of 2021. Only eight inputs had a dollar value applied, as SROI conventions state that time spent by main beneficiaries on usual activities should not be assigned a financial value [17]. To avoid this, no costs were attributed to delivery of supportive care inputs by healthcare professionals for work which would form a component of their usual roles; however, health professional wages associated with the delivery of supportive care inputs were instead assigned under the healthcare service management stakeholder groups in the SROI model. Likewise, time spent during attendance for supportive care services by patients was not costed, but ancillary costs (for example, transportation costs or fees associated with attending supportive care services) were documented for each input to capture out-of-pocket costs. When more than one valuation could be assigned to an input, the higher cost was chosen (e.g. per Assumption 1, Table 2) to ensure the analysis yielded the most conservative return on investment ratio. A 3% discounting rate was applied to account for inflation over the five-year time forecast period and ensure supportive care inputs were not over or undervalued [18].

Outcomes, indicators, and financial proxies: In order to determine the monetary value of an outcome, firstly an indicator and then a financial proxy must be assigned, as social outcomes often do not have an established market value or an inherent/agreed measure of benefit. SROI methodology places emphasis on generating indicators from stakeholders to inform and assess the value of change that has occurred. Data from patients, healthcare professionals, and healthcare services management staff were used to determine key outcomes, and how much of the outcome they attributed to a specific supportive care activity.

Stakeholder data were then supplemented with evidence from peer-reviewed literature in this SROI study (Table 4). Where an outcome was experienced by more than one group of stakeholders, it was only included for the stakeholder group who articulated the potential for most change or benefits, to avoid double-counting. For example, the outcome “symptoms and side effects of treatment are avoided, mitigated, or self-managed” was identified by all stakeholder as an outcome, however, this outcome was assigned only to the patient stakeholder group as they received the most direct benefit.

**Table 4:** Evidence consensus excerpt showcasing input development and valuation for equitable and coordinated care for all stakeholders

Supportive Care Need: Equitable and Coordinated Care				
**Published Evidence**	**Stakeholder**	**Consultation Evidence**	**Stakeholder Inputs**	**Valuation**
Care coordinators are specialised health care workers who aim to address barriers to healthcare access and manage complex health conditions; thereby reducing symptom burden, healthcare costs, and the risk of medical errors [19, 20]. This is particularly important for lung cancer patients given the complexity of their care [21, 22]. Care coordinators have been shown to be essential to efficient transfer and sharing of information between the many health professionals that constitute a cancer patients’ health care team and are important to direct timely referrals to address supportive care needs [23].	Patients	*“…One service that I did have that was wonderful was a nurse, a nurse sort of a nurse advisor, and when I was having a treatment…I could ring her any time during the week if I had problems or queries with side effects, and I did have problems, and I rang her and she was wonderful” – Patient (18) at Health Service A*	People with lung cancer interact with a healthcare professional or service who has designated responsibility, and who they can contact for supportive care needs identification and care coordination	**$13.1 per trip** Average cost of public transport daily fare ($9.00) and Average cost of parking at an urban health service and car cost for 10 km drive
	Healthcare Professionals	*“…The only strengths we have really are the nurse practitioners … they’re the ones that refer typically ‘cause they make the assessments, it’s their role in our system to do the supportive care screening” - Health Professional (3) at Health Service A*	Specialised, dedicated healthcare professionals provide coordinated care, from supportive care screening and referrals, to overarching cancer care coordination	**$0** Assisting patients navigating the social welfare system will be conducted as part of usual care delivery. No direct costs will be incurred by individual healthcare professionals.
	Healthcare service Managers	*“…I mean I think care co-ordination works best if it’s clinical, so liaison nurses would be the kind of the gold standard group that we would see involved in this, really having somebody to be able to contact” - Health Professional (1) at Health Service B*	Cost of professional development of a registered nurse to become a clinical coordinator and perform the role	**$23 757 per annum** 0.25 of a social worker’s FTE is reallocated to professional development and the delivery of a targeted supportive care service for lung cancer patients. The wage of the social worker was costed as a Grade 3 A allied health professional wage ($98 987.2)

This decision was taken to ensure that no outcomes were ‘double-counted’ (that is, benefits are not counted twice for the same outcome). All 34 outcomes were assigned indicators for valuation. Both subjective indicators self-reported by stakeholders (such as improved role functioning) and objective indicators (such as acute healthcare utilisation) were identified through stakeholder consultations and published literature.

Once indicators were established, financial proxies for each indicator could then be calculated using a variety of techniques. Market-based financial proxies were used to demonstrate the cost savings of avoiding emergency department presentations and hospital admissions, as well as the cost-savings of an avoided primary care appointment. Increased income generated valuations were used to calculate wages of healthcare professionals against specific activities using data routinely published by the Australian Bureau of Statistics (ABS) [24, 25] and the wages of Victorian health professionals working in public tertiary healthcare services [26–28]. The Value of Statistical Life (VSL) was used to ascertain the benefits of reducing the risk of death. The Australian value of a statistical life year (VSLY), $213 000 in 2019 dollars [29], was adjusted to calculate the benefit of reducing the risk of injury, disease, or disability. Where existing proxies were not available, monetary valuation of indicators were developed using a revealed preference technique, where stakeholder information was used to infer economic value of an associated impact [30] or contingent valuation [10]. Considerations have been incorporated in light of these valuation procedures in the interpretation of results and reporting of limitations.

#### Stage 4: establishing impact

Each of the monetary values for all outcomes were then adjusted to reflect what could reasonably be considered attributable to, or the impact associated with the provision supportive care. Specifically, these factors allow for determination of: how much of the outcome could have occurred without supportive care (deadweight), substitution effects between outcomes, and finally reduction in degree of attribution for each outcome relative to supportive care over time (relevant calculations are summarised in Table 5). The application of each of these adjustment calculations acknowledges the substantial heterogeneity of needs and experiences within the lung cancer population, as well as throughout an individuals’ cancer journey. Adjusted values were then used in the SROI calculation.

**Table 5:** Attribution, deadweight, displacement and drop-off

Attribution	Attribution was calculated by estimating units for each valued outcome per patient (informed by the impact map), stakeholders were involved in these calculations, and provided expertise in estimating, for example, how many unnecessary emergency room presentations could be avoided for a patient with lung cancer over a five period if they were receiving quality supportive cancer care [11, 31] Values were then calculated on a per unit basis, per capita over a one-year period, and a five-year period.
Deadweight	Deadweight was calculated as a percentage of the outcome that could have occurred if the patient had not received any supportive care, this percentage of the monetary value was then deducted from each value for each year (after the first year of implementing supportive cancer care) [31]
Displacement	Displacement was considered in the SROI but as the alternative to supportive care, in the context of the forecast was no supportive care, and factors displacing outcomes in the SROI would likely include treatment effects for patients, and the natural history of lung cancer disease, it was not possible to accurately apply an adjustment for this.
Drop-off	Finally, drop-off was calculated as a percentage of the outcome that would reduce in value over time. This estimation was informed by the extent of variability in an outcome based on individual patient characteristics, and disease trajectory over a one-year and five-year period. For outcomes where variability was known to be high (based on stakeholder views and the evidence), a 50% drop-off rate was applied. Where variability was likely but known not to be high, a 20% drop-off rate was applied. For all other patient outcomes, a 10% drop-off rate was applied. Healthcare system cost savings were excluded from drop-off rates because they were absolute values and discounting process captured effects of inflation.

#### Stage 5: calculating the SROI

The SROI ratio which describes the value generated, or ‘return on investment’, was calculated in Stage 5, using the sum of the net present values of stakeholder assigned benefits (outcomes) divided by the total cost (net present values) of investment of cancer supportive care activities (inputs) for each patient time-forecast.

$$\frac{{\varvec{V}}{\varvec{a}}{\varvec{l}}{\varvec{u}}{\varvec{e}}\ {\varvec{o}}{\varvec{f}}\ {\varvec{b}}{\varvec{e}}{\varvec{n}}{\varvec{e}}{\varvec{f}}{\varvec{i}}{\varvec{t}}{\varvec{s}}\ (\mathrm{\$})}{{\varvec{V}}{\varvec{a}}{\varvec{l}}{\varvec{u}}{\varvec{e}}\ {\varvec{o}}{\varvec{f}}\ {\varvec{I}}{\varvec{n}}{\varvec{v}}{\varvec{e}}{\varvec{s}}{\varvec{t}}{\varvec{m}}{\varvec{e}}{\varvec{n}}{\varvec{t}}\ \left(\mathrm{\$}\right)}={\varvec{S}}{\varvec{R}}{\varvec{O}}{\varvec{I}}\ {\varvec{R}}{\varvec{a}}{\varvec{t}}{\varvec{i}}{\varvec{o}}$$

A ‘combined’ SROI ratio was calculated for each time forecast (one-year time-forecast, and five-year time-forecast), that captures all outcomes benefiting both the patient and the healthcare system. Next, individual ratios were calculated to describe the outcomes which specifically benefit the healthcare system (as described by health system representative stakeholders: health professionals and healthcare managers), and those outcomes experienced by patients or “patient benefits”. Importantly, these individual ratios are not intended to be interpreted or cited independently to the combined SROI; as investment would be required by both parties if the hypothetical model were to be applied in ‘real life’. Inclusion of individual ratios is solely to demonstrate that both patients and the healthcare system would experience benefit if cancer supportive care services were to be routinely co-designed with key stakeholders and implemented in acute care settings.

A sensitivity analysis was then undertaken to test the influence of expected variability in outcomes to patients associated with cancer supportive care on ratio results (supplemental materials).

Stage 6: Reporting, using and embedding Full finding draft reporting was circulated to specifically curated group of experts comprising: health economics/SROI methodology; policy-makers, consumer advocates, data and healthcare quality and safety. Once the first round of feedback was integrated, findings/reporting was then circulated to national and international experts in lung cancer and SROI methods for feedback and review. Final results were then disseminated to key stakeholders, academic and policy audiences, and the lay community.

## Results

### SROI ratio: benefit to both patients and the healthcare system

The estimated annual costs per capita to deliver optimal supportive care at an individual healthcare service for an individual patient (inclusive of both healthcare system and patient investment) is modelled by this study at AUD$22 676. The healthcare system contributes 92% of investment, and patients contribute the remaining 8% as out-of-pocket costs. The value generated from these modelled supportive care service investments in a one-year period for both the healthcare system and the patient combined is AUD$200 800, resulting in an SROI ratio of 1:9 That is, for every dollar invested in supportive care, AUD$9 return is obtained when savings to the healthcare system and benefits to the patients are combined. Importantly, these benefits can only be achieved if all 43 inputs are invested in, and available for each individual patient.

Notably, value generated from these investments increases cumulatively over time. At five-years, the annual investments result in greater value generated for both the patient and the healthcare system: AUD$1 186 422, SROI ratio of 1:11. Therefore, over five years, for every dollar invested in supportive care, AUD$11 return is obtained when savings to the healthcare system and benefits to the patients are combined.

### SROI ratio: benefit to the healthcare system

The value generated to the healthcare system, relative to investments made in supportive care (excluding costs borne by the patient), over a one-year period is AUD$103 076. The total investment per capita that the healthcare system is required in this model is AUD$20 915 per annum, generating a SROI ratio of 1:5. Investments and benefits change slightly when projected over five years, with the total annual cost of delivering supportive care per patient over five years was estimated at AUD$98 658 compared with the value generated to the healthcare system, AUD$671 002, achieving a larger SROI ratio of 1:7.

### SROI ratio: benefit to patients

People with lung cancer in the forecasted model are required to spend a maximum of 8% out-of-pocket costs (AUD$1 762 per annum) towards the overall investment in supportive care. The social value generated to patients based on this investment upon receipt of cancer supportive care services detailed in the model is estimated at AUD$97 725 generating at a SROI ratio of 1:56. As with the healthcare system, value generated through patient investment in supportive care services increases over time, with a five-year total of AUD$535 408 and SROI ratio of 1:64.

## Discussion

This study is the first in the world to model the prospective return on investment associated with implementation of cancer supportive care services for lung cancer patients. Importantly, results demonstrate that a significant return on investment can be achieved, and shared by both patients and the healthcare system, if evidence-based cancer supportive care services are integrated as part of comprehensive oncology care. Our model describes the cancer supportive care services and human resources required for investment in order to achieve optimal health and quality of life outcomes for lung cancer patients, with clear value and cost-savings achieved. Despite clear utility and policy-to-practice application, the field of SROI methodology in economic evaluation of public healthcare interventions is in its infancy [10] Our study provides a pragmatic template for the use of this methodology to gather robust data regarding value and return on investment for value-based patient-centred models of care. Furthermore, evidence generated by this SROI analysis aligns with core elements of the value-based healthcare system model articulated by Lee et al. [32]. Value-based healthcare asserts that re-design of services around what patients need and value will result in improved outcomes, cost-savings, and greater return on investment [32].

Patients, healthcare professionals and managers, supported by a large body of literature, identified five key areas within which key activities associated with supportive care delivery should operate:: screening to determine individual patient need, information to empower patients to understand and self-manage their care, coordination of care that is equitable and culturally safe, and access to services which assist patients in managing financial toxicity, as well as those that assist with the management of anxiety and depression. When these needs are met through provision of comprehensive supportive cancer care, significant social value and return on investment is demonstrated for both patients and the healthcare system across a range of outcomes, from improved access and adherence to treatment, to reduced ED settings, to improved health outcomes and survival. For every dollar invested, AUD$9 return is obtained when savings to the healthcare system and benefits to the patients are combined, with social value increasing to AUD$11 over time (at five years). This finding is congruent with broader evidence of the cost-benefits associated with the implementation of preventative and early intervention activities compared with acute response interventions [7]. The added value demonstrated over time presents an economic imperative to support the investment into quality supportive care at a system level. Importantly, lung cancer in Australia is currently, and project to remain, the fifth most commonly diagnosed cancer in Australia, with estimates of 14,529 new cases diagnosed in 2022 alone [33]. Globally both incidence and mortality is set to rise [1]. Investment in high-value services will protect the integrity of healthcare systems both for those suffering now, and into the future as healthcare usage is projected to increase [32].

As cancer incidence grows globally, evidence is required by policy makers and healthcare system planners to design cost-effective models of care which achieve optimal patient outcomes. Notably, investment in cancer supportive care for lung cancer patients is affordable and provides value for money for public healthcare systems. Further, investment in supportive care workforce and services outlined in this study can be leveraged to other cancer types with potential to achieve efficiency and impact through economies of scale.

### Limitations

Importantly, the patient SROI ratio is high due to development of the cancer supportive care model in-line with universal health coverage in New Zealand, Canada, United Kingdom and Australia. The applicability of this model in healthcare systems financed through other mechanisms, such as private health insurance or social health insurance is unknown. Health professional stakeholders comprised predominantly oncologists over nursing or allied health professionals, due to COVID-related restrictions impacting recruitment. Given the scarcity of SROI literature in the health sector, no comparison studies were available for review, limiting some aspects of the analysis, including considering displacement.

## Conclusion

Data from this SROI study robustly demonstrate the potential of quality supportive care to prevent or mitigate harms caused by unmet need that impact health outcomes and/or quality of life. Services which improve the efficiency and cost-effectiveness of healthcare service delivery for increasingly resource constrained healthcare services and systems are vital. The findings from this study make an important contribution to guide policy and advocacy recommendations to effect outcomes.

## Supplementary Information

**Additional file** **1.**

## Data Availability

The datasets generated during the current study are available from the corresponding author on reasonable request and HREC approval conditions.

## Abbreviations

SROI: Social Return on Investment
HREC: Human Research Ethics Committee
SD: Standard deviation
NSCLC: Non-small cell lung cancer
ABS: Australian Bureau of Statistics
VSL: Value of Statistical Life
VSLY: Value of a statistical life year
