# Supplementary material for: Social return on investment economic evaluation of supportive care for lung cancer patients in acute care settings in Australia
Source: BMC Health Serv Res. 2022 Nov 23;22:1399. doi: 10.1186/s12913-022-08800-x (PMC9685972; doi:10.1186/s12913-022-08800-x)
Supplement: Supplementary file 1 — Additional file 1. [file 12913_2022_8800_MOESM1_ESM.zip › Supplementary materials_Impact Map for SROI.pdf]

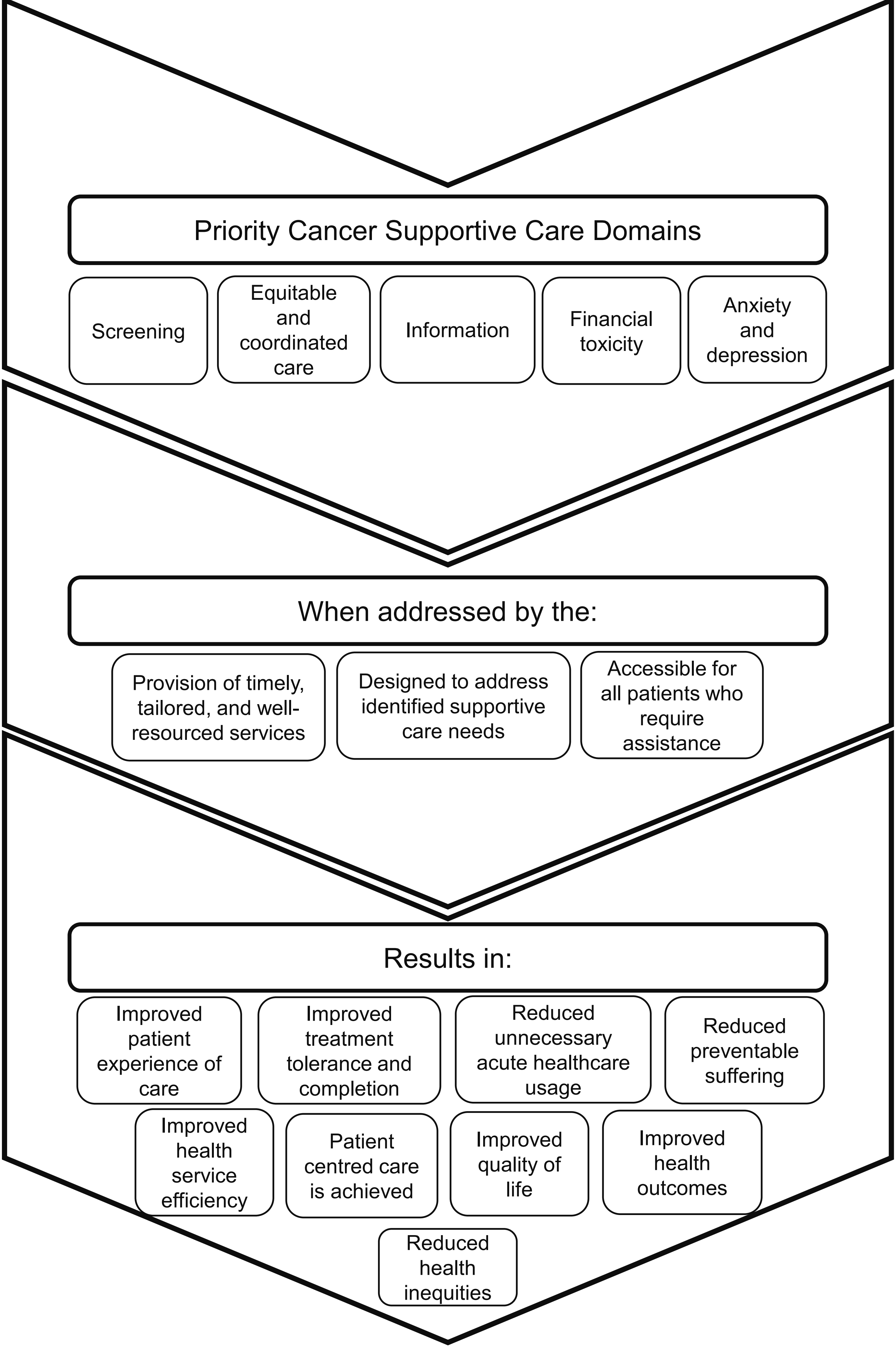

Priority Cancer Supportive Care Domains
Screening
Equitable and coordinated care
Information
Financial toxicity
Anxiety and depression
When addressed by the:
Provision of timely, tailored, and well-resourced services
Designed to address identified supportive care needs
Accessible for all patients who require assistance
Results in:
Improved patient experience of care
Improved treatment tolerance and completion
Reduced unnecessary acute healthcare usage
Reduced preventable suffering
Improved health service efficiency
Patient centred care is achieved
Improved quality of life
Improved health outcomes
Reduced health inequities
