# Supplementary material for: Social return on investment economic evaluation of supportive care for lung cancer patients in acute care settings in Australia
Source: BMC Health Serv Res. 2022 Nov 23;22:1399. doi: 10.1186/s12913-022-08800-x (PMC9685972; doi:10.1186/s12913-022-08800-x)
Supplement: Supplementary file 1 — Additional file 1. [file 12913_2022_8800_MOESM1_ESM.zip › Supplementary materials_Search Terms.docx]

# Supplementary materials

**Search strategy for scoping review**

Search terms used comprised:

descriptors of population [lung cancer AND patients AND prevalence]

AND phenomena of interest [supportive care OR need OR unmet need OR patient needs OR value OR stigma OR disparities OR inequities OR symptoms OR burden OR cluster OR adverse]

OR descriptors of intervention received [treatment OR chemotherapy OR systemic therapy OR immunotherapy]

OR descriptors of study design [interview OR survey OR review]

OR descriptors of evaluation [experiences OR perspectives OR patient reported outcomes]

OR descriptors of research type [quantitative OR review OR analysis OR synthesis]
