# Supplementary material for: Social return on investment economic evaluation of supportive care for lung cancer patients in acute care settings in Australia
Source: BMC Health Serv Res. 2022 Nov 23;22:1399. doi: 10.1186/s12913-022-08800-x (PMC9685972; doi:10.1186/s12913-022-08800-x)
Supplement: Supplementary file 1 — Additional file 1. [file 12913_2022_8800_MOESM1_ESM.zip › Supplementary materials_Sensitivity Analysis.docx]

# Supplementary materials

**Sensitivity Analysis**

A sensitivity analysis was conducted using estimated changes in patient spending on leisure activities that they might engage in due to an improved sense of health and wellbeing to see how changes in patient spending could affect the combined healthcare and patient SROI ratios calculated for both the one- and five-year cohorts. When the ratios incorporating patient spending on leisure activities were compared to the original ratios, the differences were negligible: 1:8 (for patients engaged in leisure activity spending) versus 1:9 (patients not engaged in leisure activity spending) for benefits generated over one year, and 1:11 versus 1:11 for benefits for the same groups generated over five years. When the ratio figures are rounded to the nearest whole number there is a slight difference reflected in the value generated for the one-year cohort, and no difference in value generated for the five-year cohort, demonstrating that the benefits generated by this model of optimal supportive care are likely to be stable in response to changes in patient spending. However, it would be remiss not to acknowledge that this is likely the case because of a low proportion of investment required on the part of the patient. Ratios may be less stable had variation in the quality of supportive cancer care been able to be incorporated in the sensitivity analysis, but due to a lack of evidence this was not possible.
